# IRF2 inhibits ZIKV replication by promoting FAM111A expression to enhance the host restriction effect of RFC3

**DOI:** 10.1186/s12985-021-01724-8

**Published:** 2021-12-20

**Authors:** Kai Ren, Ya Zhu, Honggang Sun, Shilin Li, Xiaoqiong Duan, Shuang Li, Yujia Li, Bin Li, Limin Chen

**Affiliations:** 1 Institute of Blood Transfusion, Chinese Academy of Medical Sciences and Peking Union Medical College, 26 Huacai Road, Chengdu, 610051 China; 2 The Seventh Affiliated Hospital, Sun Yat-sen University, Shenzhen, China; 3 The Joint Laboratory on Transfusion-Transmitted Diseases (TTDs) Between Institute of Blood Transfusion, Chinese Academy of Medical Sciences and Nanning Blood Center, Naning Blood Center, Nanning, China; 4 Toronto General Research Institute, University of Toronto, Toronto, Canada; 5 Shanghai University of Medicine and Health Sciences, Shanghai, China

**Keywords:** ZIKV, IRF2, Jak/STAT signaling pathways, FAM111A, RFC3

## Abstract

**Background:**

Although interferon regulatory factor 2 (IRF2) was reported to stimulate virus replication by suppressing the type I interferon signaling pathway, because cell cycle arrest was found to promote viral replication, IRF2-regulated replication fork factor (FAM111A and RFC3) might be able to affect ZIKV replication. In this study, we aimed to investigate the function of IRF2, FAM111A and RFC3 to ZIKV replication and underlying mechanism.

**Methods:**

siIRF2, siFAM111A, siRFC3 and pIRF2 in ZIKV-infected A549, 2FTGH and U5A cells were used to explore the mechanism of IRF2 to inhibit ZIKV replication. In addition, their expression was analyzed by RT-qPCR and western blots, respectively.

**Results:**

In this study, we found IRF2 expression was increased in ZIKV-infected A549 cells and IRF2 inhibited ZIKV replication independent of type I IFN signaling pathway. IRF2 could activate FAM111A expression and then enhanced the host restriction effect of RFC3 to inhibit replication of ZIKV.

**Conclusions:**

We speculated the type I interferon signaling pathway might not play a leading role in regulating ZIKV replication in IRF2-silenced cells. We found IRF2 was able to upregulate FAM111A expression and thus enhance the host restriction effect of RFC3 on ZIKV.

## Introduction

ZIKV (zika virus) belongs to the flaviviridae family, and can transmit from mosquitoes to humans to cause microcephaly and Guillain–Barré syndrome in most severe cases [1–3]. ZIKV genome is a single positive stranded RNA consisting of approximately 11 kb nucleotides, which encodes three structural proteins (C, PrM and E) and seven non-structural proteins (NS1, NS2A, NS2B, NS3, NS4A, NS4B and NS5) [4]. ZIKV infection has become one of the serious global public health threat since its first outbreak in Yap Island in 2007 [5]. Neither vaccine nor specific antiviral therapy is available up till now. Therefore, it is critical to explore the underlying mechanism on how the host responds to ZIKV infection.

The Interferon regulatory factor (IRF) family comprises nine members, including IRF1, IRF2, IRF3, IRF4, IRF5, IRF6, IRF7, IRF8 and IRF9 [6]. They participate in a variety of biological processes including antiviral inflammation, proliferation, apoptosis, and maturation of immune cell, and therefore can participate in both immunity and oncogenesis [7, 8]. Among the nine members, IRF1, IRF2, IRF3 and IRF7 are the master regulator of transcriptional activation of type I interferon genes [9]. When virus infect host cell, IRF3 and IRF7 were activated with phosphorylation and then induce Type I Interferons expression [10]. Type I Interferons (mainly IFNα/β) induce the expression of a few hundred interferon-stimulated genes (ISGs) by activating the Jak-STAT signaling pathway to exert its anti-viral effect [11]. The anti-viral function of many ISGs in multiple flavivirus infections, including ZIKV, DENV, WENV, JEV, has been largely confirmed [12–15]. Type I Interferons signaling pathway play a key role in ZIKV-infected A549 cells and brain.

Because viral infection and upregulate IFN-β can induce IRF1 and IRF2 expression, when Type I Interferons signaling pathway is overactivated, IRF2, which acts as a transcriptional repressor through competing with IRF1 for binding to the IFNβ promoter region or Interferon-sensitive response element (ISRE) of the ISGs promoter subzone, negatively regulates IFNβ signal and interferon stimulated gene factor 3 (ISGF3)-mediated gene induction [9, 16–19]. Type I interferon signaling pathway plays a important role in the process of ZIKV infection of a549 cells and brain [12, 20]. However, whether IRF2 affects ZIKV replication through regulating ISGs are still unclear.

In addition to negatively regulate type I IFN signaling pathway, IRF2 has also been reported to affect dsDNA virus replication by regulating Family with sequence similarity 111 member A (FAM111A) and replication factor C subunit 3(RFC3) [21]. As a chromatin-related protein sharing homology with trypsin-like peptidase, FAM111A interacts with proliferating cell nuclear antigen (PCNA) and binds to SV40 large T antigen, acting as a host restriction factor for SV40 [22, 23]. Replication factor C (RFC) plays an important role in DNA replication, DNA damage repair, and check point control of cell cycle. RFC3 is one of the five subunits of RFC and can load PCNA onto DNA at template primer junctions [24]. However, whether IRF2 regulates ZIKV replication through FAM111A and RFC3 remains to be determined. In this study, we therefore investigated the role of IRF2 in ZIKV replication and the potential underlying mechanism. We found that IRF2 inhibited ZIKV replication through regulating IRF2-FAM111A-RFC3 axis.

## Materials and methods

### Cell culture and infection with ZIKV

A549 cells (human non-small-cell lung cancer cell line) were purchased from West China Hospital of Sichuan University. Mutant U5A cells were derived from the parental human fibrosarcoma cells (2fTGH cells) and had IFNARI gene-deficient for a component of the IFNα/β receptor were kindly provided by Dr. Wenyu Lin (Harvard Medical School, USA) [25, 26]. A549 cells, 2FTGH cells and U5A cells were grown in Dulbecco′s Modified Eagle′s Medium (DMEM), supplemented with 10% fetal bovine serum (FBS), 100 μg/mL penicillin and 100 μg/mL streptomycin sulfate. Cells were cultured in a 5% CO2 humidified incubator at 37 °C. ZIKV (GZ01 strain) was generously provided by professor Chengfeng Qin (Institute of Microbiology and Epidemiology, China) and was propagated in mosquito C6/36 cells as described in previous publication [27]. For virus infection, cells were infected with ZIKV at a multiplicity of infection (MOI) of 0.5 with serum-free and antibiotic-free medium and incubate for 4 h. Cells were then washed with PBS for 3 times and re-fed with normal medium.

### RNA isolation and quantitation by RT-qPCR

Total RNAs were extracted using Trizol reagent (Invitrogen, USA) according to the manufacturer′s instructions. cDNA was synthesized using the First Strand cDNA Synthesis Kit (Bio-rad, USA). Real-time PCR was performed with Fast start Universal SYBR Green Master Mix (Roche, USA). Normalization and quantitation were performed using GAPDH RNA and the ΔΔCt method. The selected mRNA real-time PCR primers were listed in Table 1.

**Table 1:** Real-time PCR primers

Gene name	Forward primer (5′–3′)	Reverse primer (5′–3′)
GAPDH	GCCTCCTGCACCACCAACTG	ACGCCTGCTTCACCACCTTC
IRF2	ATTTGCCAAGTTGTAGAGG	CTATCAGTCGTTTCGCTTT
IFIT1	GCAGCCAAGTTTTACCGAAG	GCCCTATCTGGTGATGCAGT
FAM111A	CTTCACAAAAAGGGCGCAA	ATCAACTGGCTGGGTGCTTT
RFC3	GCCTGCAGAGTGCAACAATA	TCAAGGAGCCTTTGTGGAGT
ZIKV NS5	GCAGAGCAACGGATGGGAT	ATGGTGGGAGCAAAACGGA
ISG15	CGCAGATCACCCAGAAGATT	GCCCTTGTTATTCCTCACCA

### Protein quantification and western blot assay

The cells were washed 3 times with PBS, and then lysed by adding 100 μL of Radio Immunoprecipitation Assay (RIPA) (Beyotime, China) containing PMSF. Cell lysates were centrifugated and protein concentration was quantified with the Protein Assay Kit (Beyotime, China). Protein samples were separated by SDS–PAGE gels and transferred to the PVDF membranes (Millipore, USA). The membranes were blocked using 5% bovine serum albumin (BSA) and then incubated with the specific primary antibody in 4 °C for 16 hours. The primary antibodies included rabbitanti-IRF2 (Proteintech, China), mouse anti-ZIKV NS1 (GeneTex, USA), rabbitanti-RFC3 (Proteintech, China), rabbitanti-IRF2(Abcam, UK) and mouse anti-GAPDH (GeneTex_, USA). The secondary antibody used was HRP-labeled goat anti-mouse IgG or anti-rabbit (Beyotime, China). The protein bands were detected using the ECL Western Blotting Analysis System (Mllipore, USA) on Image Quant LAS 4000 mini (GE, USA).

### RNA interference and plasmid transfection

Specific small interfering RNAs (siRNAs) were purchased from Sangon company (shanghai, China). The sense and antisense sequences of the siRNAs are listed in Table 2. pIRF2 (plasmid IRF2) and pEmpty (empty control) were purchased from vigene biosciences company (Shandong, China). Double-stranded siRNA and plasmid were transfected into cells with RNAiMax (Invitrogen, USA) according to the manufacturer′s instruction.

**Table 2:** RNA interference sequences

siRNAs	Sense (5′–3′)	Antisense (5′–3′)
siNC	UUCUCCGAACGUGUCACGUT	ACGUGACACGUUCGGAGAATT
siIRF2	GCAAUCCGGUGCCUUACAAT	UUGUAAGGCACCGGAUUGCTT
siFAM111A	GGUCAAUGUGUAAGGG	UCACCCUUACACAUUGACC
siRFC3	AAGUAACUACCACCUUGAA	UAACUUCAAGGUGGUAGUUA

### Statistical analysis

Data from all cell-based assays were expressed as Mean ± standard deviation (SD). Student's t-test was used to assess statistical significance of differences. We used one-way analysis of variance (ANOVA) to more than one variable. The chart was obtained using the GraphPad Prism 5.0 software package (GraphPad Prism Software, La Jolla, CA, USA). *P*-values less than 0.05 were considered statistically significant.

## Results

### IRF2 expression is increased in ZIKV-infected A549 cells

**Fig. 1:**
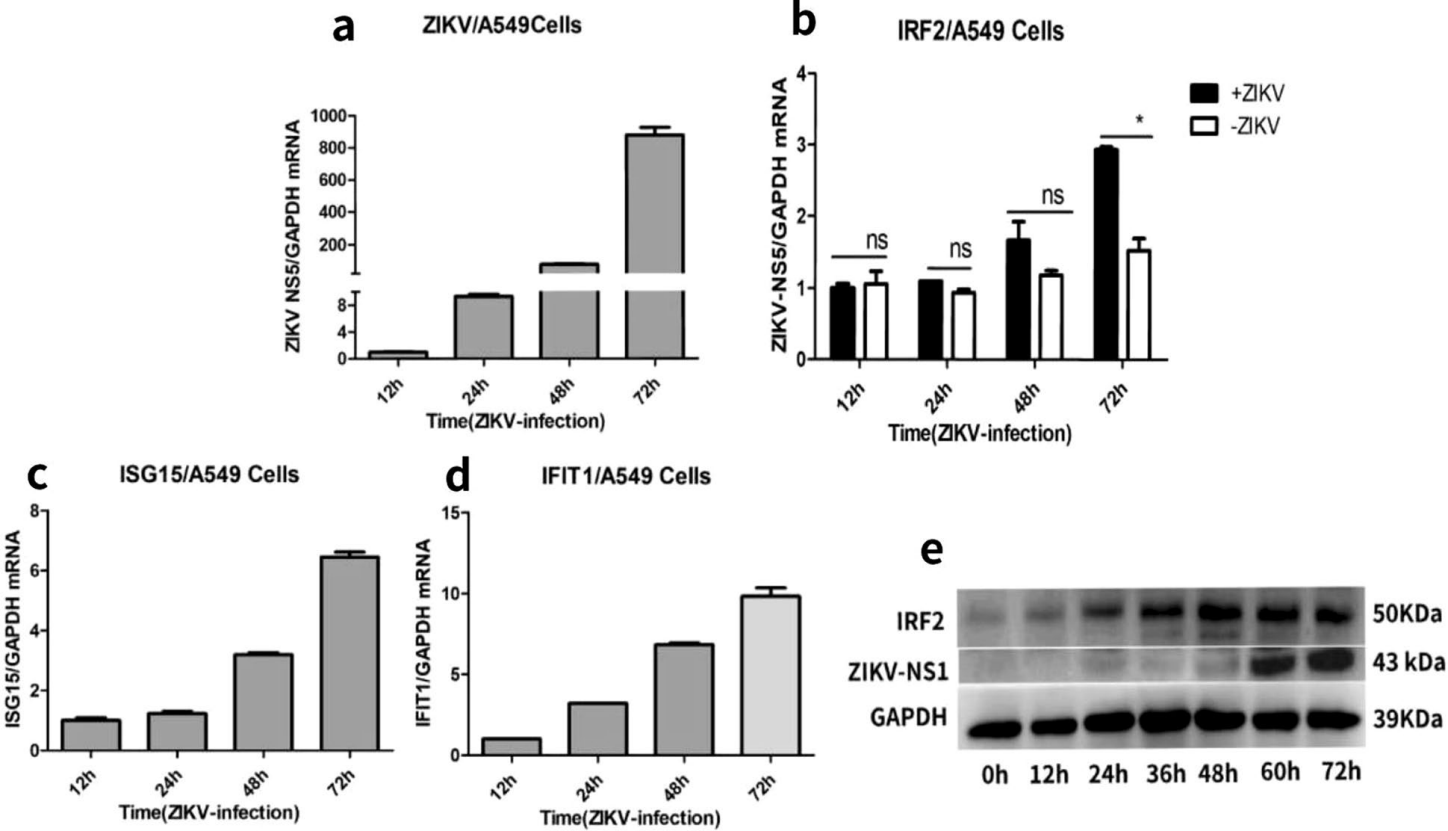
ZIKV replicates and IRF2 is induced in A549 cells. A549 cells were infected with ZIKV (MOI = 0.5) or left uninfected and total cellular RNAs were extracted at different hours-post-infection (hpi). RNA levels of ZIKV NS5 (**a**), IRF2 (**b**), ISG15 (**c**) and IFIT1 (**d**) were measured by RT-qPCR. Protein levels of ZIKV-NS1 and IRF2 were measured by western blots (**d**). Data were presented as mean ± standard deviation (SD) representative of at least three independent experiments

To investigate whether the expression levels of IRF2 were changed following ZIKV replication by time, A549 cells were infected with ZIKV at a MOI of 0.5. mRNA and protein expression levels of ZIKV and IRF2 were measured at 12, 24, 48 and 72 h-post-infection (hpi) by RT-qPCR and western blots. We calculated the relative expression of ZIKV RNA and IRF2 by normalizing ZIKV NS5 and IRF2 to GAPDH, respectively. As shown in (Fig. 1a, b, e), ZIKV can steadily replicate in A549 cells, and compared with the uninfected control, IRF2 expression is gradually increased from 12 to 72 h post-infection. In addition, to assess effect of mainly antiviral factor (ISGs) in ZIKV-infected A549 cells, we examine expression levels of ISG15 and IFIT1 at different time (Fig. 1c, d). These results indicated that ZIKV can infect and replicate steadily in A549 cells and IRF2, ISG15 and IFIT1 expression was increased upon ZIKV infection.

### Knocking down of IRF2 promoted ZIKV replication through type-I IFN signal pathway-independent way

**Fig. 2:**
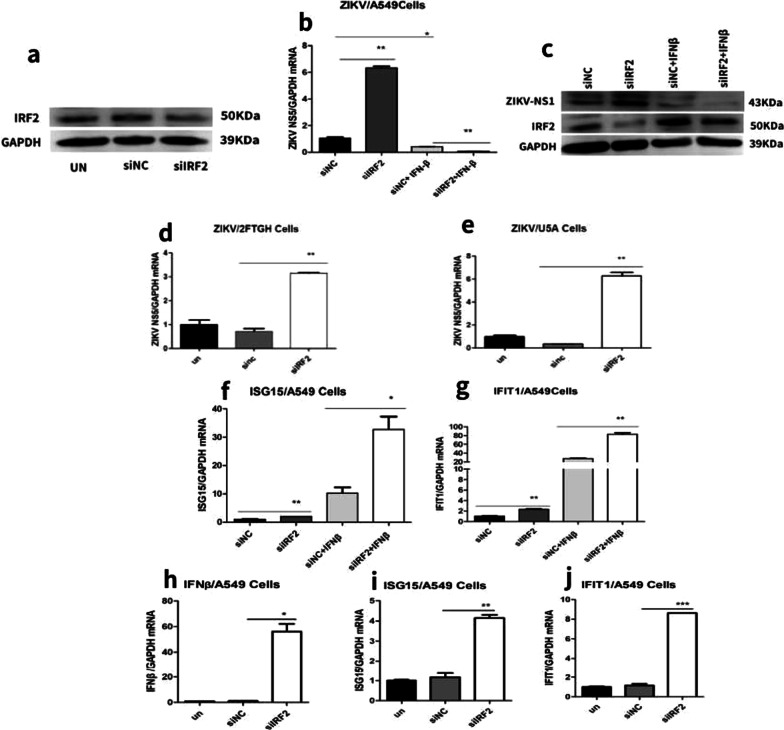
Knockdown of IRF2 promoted ZIKV replication independent of type I IFN signaling. A549 cells were transfected with siIRF2 or siNC and infected with ZIKV at MOI of 0.5. After 24 h, IFNβ was added to appropriate wells to a final concentration of 100 IU/mL. Total RNAs and proteins were extracted 24 h later, and selected gene expression was tested by RT-qPCR (**b**, **f**, **g**) and western blot (**a**, **c**). The relative ZIKV production as affecting of siIRF2 and IFNβ were significantly different (siIRF2*IFNβ, *P* = 0.000, F = 919.4). The relative ISG15 production as affecting of siIRF2 and IFNβ were significantly different (siIRF2*IFNβ, *P* = 0.012, F = 19.1). The relative IFIT1 production as affecting of siIRF2 and IFNβ were significantly different (siIRF2*IFNβ, *P* = 0.000, F = 224.4). 2FTGH and U5A cells were transfected with siIRF2 or siNC and infected with ZIKV (MOI = 0.5). After 24 h, mRNA expression of ZIKV-NS5 was examined with RT-qPCR (**d**, **e**). siIRF2 or siNC were transfected into A549 cells, IFNβ, ISG15 and IFIT1 RNA levels were extracted and examined with RT-qPCR at 24 h later (**h**, **i**, **j**). Data was shown as mean ± standard deviation representative of at least three independent experiments (*t* test, *P < 0.05; **P < 0.01)

RNA interference was employed to dissect the role of IRF2 in ZIKV replication. We found IRF2 siRNA markedly knocked down IRF2 protein levels (Fig. 2a) and promoted ZIKV replication both at mRNA and protein levels in A549 cells (Fig. 2b, c). As IRF2 has been reported to be a negative regulator of type-I interferon pathway, we next checked mRNA expression of two classical interferon stimulated genes, ISG15 and IFIT1 in IRF2-silenced A549 cells. We found that silencing of IRF2 indeed increased the mRNA expression of ISG15 and IFIT1 significantly (Fig. 2f, g), but seems contradictory to the stimulating effect on ZIKV because these ISGs are classical anti-viral proteins [28]. To confirm the type-I interferon pathway is intact in A549 cells, we were able to detect the significant inhibitory effect of IFNβ on ZIKV replication by treating the ZIKV-infected cells with 100IU/mL IFNβ. In addition, compared to IFNβ treatment alone, combination of IFNβ treatment and IRF2 silencing increased ISG15 (Fig. 2f) and IFIT1 (Fig. 2g) mRNA expression to a higher level and inhibited ZIKV replication more significantly both at RNA (Fig. 2b) and protein levels (Fig. 2c). as the control, we examined IFNβ, ISG15 and IFIT1 RNA levels (Fig. 2h–j). All these data pointed out the type-I IFN-activated Jak/STAT signaling is intact in A549 cells. Furthermore, we also used an IFNAR-deficient U5A cells to verify whether the stimulating effect of IRF2 silencing on ZIKV replication is dependent on the type-I IFN signaling pathway. We found that IRF2 siRNA increased ZIKV replication as shown by NS5 mRNA expression in both U5A and its parental 2FTGH cells (Fig. 2d, e). Collectively, these results indicated that IRF2 regulate ZIKV replication in an IFN-independent manner.

### IRF2 activates FAM111A expression and then enhances the host restriction effect of RFC3 on ZIKV

**Fig. 3:**
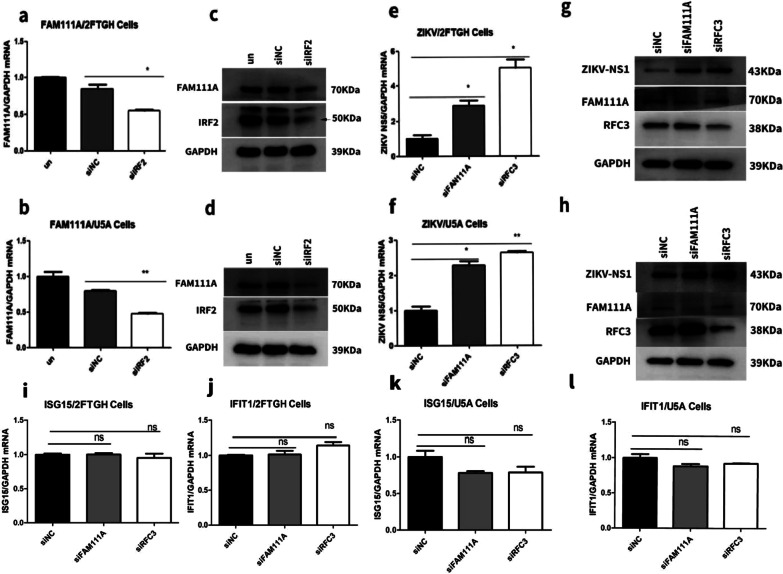
IRF2 silencing decreased FAM111A expression and knockdown of FAM111A or RFC3 promoted ZIKV replication in both 2FTGH and U5A cells. 2FTGH and U5A cells were transfected with siNC or siIRF2 for 24 h, and then the cells were infected with ZIKV at MOI of 0.5. Expression levels of FAM111A were examined both at mRNA (**a**, **b**) and protein (**c**, **d**) levels 48 h post transfection. 2FTGH and U5A cells were transfected with siNC, siFAM111A, or siRFC3 for 24 h, and then the cells were infected with ZIKV at MOI of 0.5. Expression levels of ZIKV-NS5 were examined both at RNA (**e**, **f**) and protein levels (**g**, **h**) 48 h post transfection. ISG15 and IFIT1 were examined at mRNA levels with RT-qPCR (**i**–**l**). The relative ZIKV production in 2FTGH cells between siNC and siFAM111A (*P* = 0.03, F = 15.2) or siRFC3 (*P* = 0.004, F = 68.9) were significantly different. The relative ZIKV production in U5A cells between siNC and siFAM111A (*P* = 0.002, F = 90.1) or siRFC3 (*P* = 0.001, F = 145.6) were significantly different. Data were presented as mean ± standard deviation representative of at least three independent experiments (*t* test, *P < 0.05; **P < 0.01)

Panda et al. [21] thought that IRF2 regulated FAM111A expression, which could form a complex with RFC to restrict poxviruses replication in human cells. In order to investigate whether IRF2 silencing promoted ZIKV replication by regulating the expression of FAM111A, we transfected IRF2 siRNA to 2FTGH and U5A cells and then infected with ZIKV at MOI = 0.5. As shown in Fig. 3a–d, IRF2 knockdown decreased the expression of FAM111A significantly. Furthermore, knockdown of FAM111A or subunit of RFC (RFC3) pronouncedly promoted ZIKV replication in both 2FTGH and U5A cells as shown in Fig. 3e–h. To assess whether I type IFN signaling pathway was activited by FAM111A and RFC3 knockdown, we examined ISG15 and IFIT1 in ZIKV-infected 2FTGH and U5A cells with siFAM111A and siRFC3. Figure 3i–l showed siFAM111A and siRFC3 didn't affect ISG15 and IFIT1 expression. These data indicated that IRF2 may modulate ZIKV replication by regulating FAM111A and RFC3 expression.

### Over-expression of IRF2 inhibited ZIKV replication while this inhibitory effect was abolished in FAM111A or RFC3 knockdown cells

**Fig. 4:**
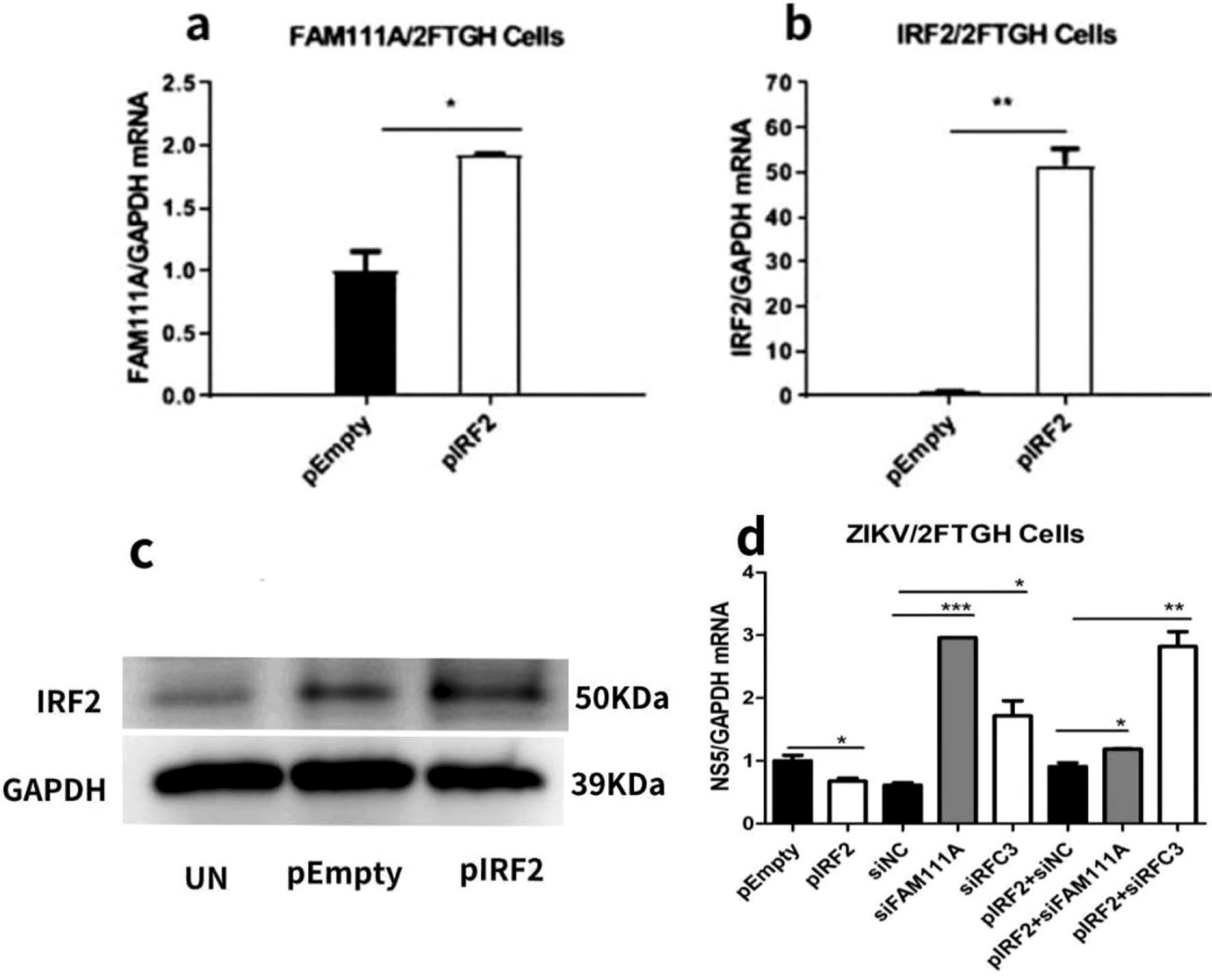
Knockdown of FAM111A or RFC3 abolished the inhibitory effect of IRF2 overexpression on ZIKV replication. The experiments were divided into 8 groups of 2FTGH cells which were transfected with pEmpty, pIRF2, siNC, siFAM111A, siRFC3, pIRF2 + siNC, pIRF2 + siFAM111A orpIRF2 + siRFC3, respectively, for 24 h. All the cells were infected with ZIKV at MOI of 0.5 and the mRNA expression levels of FAM111A (**a**), IRF2 (**b**) and ZIKV (**d**) were measured by RT-qPCR at 24 h-post-infection. 2FTGH cells was transfected with pEmpty or pIRF2 plasmid before being infected with ZIKV (MOI = 0.5) for 24 h. The relative ZIKV production in 2FTGH cells between pEmpty and pIRF2 (*P* = 0.047, F = 9.2) were significantly different. The relative ZIKV production in 2FTGH cells between siNC and siFAM111A (*P* = 0.000, F = 93.2) or siRFC3 (*P* = 0.000, F = 123.6). The relative ZIKV production in 2FTGH cells between pIRF2 and siFAM111A (pIRF2*siFAM111A, *P* = 0.000, F = 44.5) or siRFC3 (pIRF2*siFAM111A, *P* = 0.002, F = 18) were significantly different. The IRF2 protein expression (**c**) was detected with western blots (*t* test, *P < 0.05; **P < 0.01)

In order to further confirm the role of IRF2 in ZIKV replication through FAM111A and RFC3, we investigated the effect of IRF2 overexpression (Fig. 4b, c) on ZIKV replication in FAM111A or RFC3 knocked-down 2FTGH cells (Fig. 4d). We found overexpression of IRF2 increased FAM111A mRNA level by 2-fold (Fig. 4a) and inhibited ZIKV replication (Fig. 4d). However, this inhibitory effect of IRF2 on ZIKV replication was abolished in FAM111A or RFC3 knocked-down 2FTGH cells (Fig. 4d).

## Discussion

When defending against invading virus infections, host cells need to activate both innate and adaptive immune systems. Type I IFNs and the down-stream Jak/STAT signaling pathway play the utmost role in antiviral innate immunity. However, this anti-viral immunity must be balanced by a complicated network (pathways) in cells. As two antagonism transcription regulators, IRF1 and IRF2 play important roles in type I interferon-induced antiviral effect [29–31]. As previously reported, IRF1 could be induced by many cytokines such as IFNs (-α, -β, -γ), TNF-α, IL-1, IL-6, LIF and virus infection mediated by STAT and NF-κB [9]. IRF1 could bind to the IFNβ promoter region and ISRE of the ISGs promoter subzone containing IRF-E sequences to stimulate the production of IFNβ and many antiviral ISGs while IRF2 competed with IRF1 to function as a transcriptional repressor to negatively regulate the host innate antiviral immunity [9, 17–19]. The expression of IRF2 including IRF1 binding site at promoter region is inducible by transient or stable IRF1 expression [32–34]. IRF2 was up-regulated in ZIKV infection of human iris pigment epithelial cells [35]. In another study, IRF2 expression was induced during acute and latent infection [36]. Our present study also confirmed that ZIKV infection could upregulate the expression level of IRF2.

After we confirmed that IRF2 was induced in ZIKV-infected cells, we moved on to dissect the role of IRF2 in ZIKV replication. Firstly, we silenced the IRF2 expression by specific siRNA or overexpression by plasmid transfection to look at the effect of IRF2 on ZIKV replication. We found IRF2 overexpression inhibited ZIKV replication (Fig. 4d) while silencing of IRF2 stimulated ZIKV replication (Fig. 2). Previous reports that IRF2 promoted replication of caprine parainfluenza virus type 3 as a negative regulator to down-regulate the type I interferon signaling pathways [29]. This contradictory phenomenon alarmed us to look into this effect in more detail. Although silencing of IRF2 increased the expression levels of some ISGs, such as ISG15 (Fig. 2f) and IFIT1 (Fig. 2g), confirming that IRF2 functioned as negative regulator of type-I IFN signaling, knockdown of IRF2 stimulated ZIKV replication (Fig. 2). After we confirmed the type-I IFN signaling is intact in A549 cells used for this study. We hypothesized the role of IRF2 in ZIKV replication may be independent of type-I IFN signaling. Using IFNAR-deficient U5A cells, we were able to show that IRF2 knockdown increased ZIKV replication in both U5A and its parental 2FTGH cells (Fig. 2d, e), which indicated that IRF2 regulate ZIKV replication in an IFN-independent manner.

In addition to negatively regulate type I IFN pathway, IRF2 has also been reported to affect dsDNA virus replication by regulating Family with sequence similarity 111 member A (FAM111A) and replication factor C subunit 3(RFC3) [21]. Debasis Panda et al. proposed that siIRF2 may inhibit the host restriction effect of RFC3 by down-regulating the expression of FAM111A [15]. Although FAM111A and RFC3 act as nuclear localization-related factors and both played important role in DNA virus replication, whether they are involved in RNA virus such as ZIKV infection remains to be determined [21]. IRF-1 is upregulated by infection of Measles virus (MeV, RNA virus) and contributes to the growth arrest of MeV-infected human epithelial cells and efficient virus proliferation through the modulation of host cell events [37, 38]. silencing IRF2 was reported to result in growth inhibition associated with G2/M arrest as well as induction of polyploidy, differentiation and apoptosis [39]. FAM111A and RFC3 as replication fork related complex is downregulated, which will inhibit cell proliferation and survival [40, 41].

We then moved on to investigate whether IRF2 silencing promoted ZIKV replication by regulating the expression of FAM111A. As shown in Fig. 3a–d, IRF2 knockdown decreased the expression of FAM111A significantly. Furthermore, knockdown of FAM111A or subunit of RFC (RFC3) pronouncedly promoted ZIKV replication in both 2FTGH and U5A cells as shown in Fig. 3e–h. These data indicated that IRF2 may modulate ZIKV replication by regulating FAM111A and RFC3 expression. In order to further confirm the role of IRF2 in ZIKV replication through FAM111A and RFC3, we investigated the effect of IRF2 overexpression on ZIKV replication in FAM111A or RFC3 knocked-down 2FTGH cells (Fig. 4c). We found overexpression of IRF2 increased FAM111A mRNA level by 2-fold (Fig. 4a) and inhibited ZIKV replication (Fig. 4d). However, this inhibitory effect of IRF2 on ZIKV replication was abolished in FAM111A or RFC3 knocked-down 2fTGH cells (Fig. 4d). Collectively these data supported that IRF2 regulates ZIKV replication through FAM111A and RFC3.

## Conclusion

In conclusion, in this present study we identified IRF2 regulated ZIKV replication not through IFN signaling but instead through regulating with FAM111A and RFC3.

## Data Availability

The data and material generated or analyzed in this study are available upon reasonable request, and could be provided by limin_chen (limin_chen_99@yahoo.com).

## Abbreviations

IRF1: Interferon regulatory factor 1
IRF2: Interferon regulatory factor 2
ZIKV: Zika virus
FAM111A: Family with sequence similarity 111 member A
RFC3: Replication factor C subunit 3
IFNα/β: Interferon α/β
ISGs: Interferon-stimulated genes
ISG15: Interferon-stimulated gene 15
IFIT1: IFN-induced proteins with tetratricopeptide repeat
ISRE: Interferon-sensitive response element
ISGF3: Interferon stimulated gene factor 3
FAM111A: Family with sequence similarity 111 member A
RFC3: Replication factor C subunit 3
